# Increased SGLT1 expression in salivary gland ductal cells correlates with hyposalivation in diabetic and hypertensive rats

**DOI:** 10.1186/1758-5996-5-64

**Published:** 2013-10-24

**Authors:** Robinson Sabino-Silva, Maristela Mitiko Okamoto, Aline David-Silva, Rosana Cristina Mori, Helayne Soares Freitas, Ubiratan Fabres Machado

**Affiliations:** 1 Universidade Federal de Uberlândia, Instituto de Ciências Biomédicas - Área de Fisiologia e Farmacologia Av. Pará, 1720 Campus Umuruama, CEP: 38400-902 Uberlândia-MG, Brazil; 2 Department of Physiology and Biophysics, Institute of Biomedical Sciences, University of Sao Paulo, Sao Paulo, Brazil; 3 Institute of Biological Sciences and Health, Federal University of Alagoas (UFAL), Av. Lourival de Melo Mota, km 14, Campus A. C. Simões, Cidade Universitária, Maceió/AL CEP 57072-970, Brazil

**Keywords:** Salivary secretion, Xerostomia, Glucose reabsorption, Parotid, Submandibular

## Abstract

**Background:**

Oral health complications in diabetes and hypertension include decreased salivary secretion. The sodium-glucose cotransporter 1 (SGLT1) protein, which transports 1 glucose/2 Na^+^/264 H_2_O molecules, is described in salivary glands. We hypothesized that changes in SGLT1 expression in the luminal membrane of ductal cell may be related to an altered salivary flow.

**Findings:**

By immunohistochemistry, we investigated SGLT1 expression in ductal cells of parotid and submandibular glands from Wistar Kyoto rats (WKY), diabetic WKY (WKY-D), spontaneously hypertensive rats (SHR) and diabetic SHR (SHR-D), as well as in parotid glands from WKY subjected to sympathetic stimulation, with or without previous propranolol blockade. Diabetes and hypertension decreased the salivary secretion and increased SGLT1 expression in the luminal membrane of ductal cells, and their association exacerbated the regulations observed. After 30 min of sympathetic stimulation, SGLT1 increased in the luminal membrane of ductal cells, and that was blocked by previous injection of propranolol.

**Conclusions:**

SGLT1 expression increases in the luminal membrane of salivary gland ductal cells and the salivary flow decreases in diabetic and hypertensive rats, which may be related to sympathetic activity. This study highlights the water transporter role of SGLT1 in salivary glands, which, by increasing ductal water reabsorption, may explain the hyposalivation of diabetic and hypertensive subjects.

## Background

Reduced salivary flow rate has been described in diabetes [1,2], as well as in hypertension [3]; two diseases that are frequently associated. Salivary gland function is highly controlled by both the sympathetic and the parasympathetic activity [4], which may be impaired in both diabetes and hypertension.

According to the classical two-stage hypothesis of saliva production [5], a primary fluid containing plasma-like electrolyte concentration is generated by the acinar cells. The fluid is subsequently modified as it flows along the ductal system, resulting in the final, hypotonic solution that enters the mouth [6]. In this process, for the last half century, acini have been considered as the main site of water transport, with no relevant water flow in ducts [5]; however, that has never been clearly demonstrated. Besides, the study of transepithelial glucose and water fluxes in the ductal cells under pathological conditions has been ignored.

The sodium glucose cotransporter SGLT1 plays an essential role in absorbing glucose from diet in the intestine, and an adjuvant role in reabsorbing glucose from the glomerular filtrate in kidneys [7]. Recently, a powerful role of water transport has been additionally attributed to SGLT1, once the stoichiometric relationship of transport capacity was observed to be 2 Na^+^: 1 glucose: 264 H_2_O molecules [7,8].

In salivary glands, the SGLT1 protein was initially described in the basolateral membrane of acinar cell [9], where it provides glucose for the cellular metabolism [10]. Furthermore, the SGLT1 expression in acinar cells has already been described as altered in diabetes and hypertension, in a sympathetic-mediated way [10]. Recently, the SGLT1 protein has been demonstrated in the luminal membrane of ductal cells of salivary glands as well [11,12], and changes in its expression have been suggested to be involved in the diabetes-induced alterations of the salivary flow (12). However, the effects of hypertension and its association to diabetes in ductal SGLT1 expression, as well as in the resulting changes in salivary glucose concentration and the salivary flow, remain unknown.

Thus, the aims of the present study were to investigate, in ductal cells of salivary glands from diabetic and hypertensive rats: 1) the SGLT1 protein expression and subcelular localization; 2) the sympathetic activity upon the SGLT1 translocation to the luminal membrane; and 3) the correlation between changes in SGLT1 and the salivary flow.

## Methods

All experimental procedures were approved by the Ethical Committee for Animal Research of the Institute of Biomedical Sciences (University of São Paulo, Protocol #97/2007). The **s**tudy was performed in male Wistar Kyoto (WKY) and Spontaneously Hypertensive (SHR) rats (weighing ~260 g). Part of the animals was rendered diabetic (WKY-D or SHR-D) by intravenous injection of alloxan (40 mg/Kg body weight), and studied after 30 days. Five animals per group of diabetic and hypertensive rats were analyzed, plus 6 animals for acute sympathetic stimulation analysis, totaling 26 animals.

Arterial pressure was measured in unanaesthetized animals, as previously described [10]. To determine the sympathetic role upon the SGLT1 protein intracellular traffic, stimulation of the left postganglionic sympathetic nerve was performed in a group of anaesthetized WKY rats (sodium pentobarbital, 40 mg/kg, ip), in the presence or absence of a β-blocker propranolol (2 mg/kg, iv), as previously described [10]. The glands were harvested 30 min after the stimulus.

Non-stimulated salivary secretion was measured in anaesthetized rats (sodium pentobarbital, 40 mg/kg, ip) for 7 min, using four pre-weighed cotton balls inserted into the oral cavity, two underneath the tongue, and two bilaterally medial to the teeth and oral mucosa. The 7-min volume of secreted saliva was calculated by subtracting the initial from the final weight of the cotton balls, and considering that 1 mg corresponds to 1 μL. After that, still under anaesthesia, blood was sampled from the tail vein, and the parotid and submandibular glands were harvested for further immunohistochemistry analysis.

### Immunohistochemistry

Immunohistochemistry analysis was performed as previously described [12]. Briefly, tissue sections were incubated with anti-SGLT1 antibody (1:50; Chemicon International). After washing with PBS, tissues were incubated with goat antiserum against rabbit IgG tagged to Cy5 (1:200; Molecular Probes, Eugene, OR). Rhodamin-phalloidin (1:100; Molecular Probes) and Sitox Green (1:10,000; Molecular Probes) were incubated to stain F-actin and nucleous; respectively. The slides were mounted and viewed under a confocal microscope (Nikon PCM2000).

Glucose concentration was measured in plasma, saliva and urine, using the kit *Glicose Enzimática* (ANALISA Diagnostica, Belo Horizonte, MG, Brazil).

Results are presented as mean ± SEM, and statistical analysis was performed by One-Way analysis of Variance (ANOVA) and Student-Newman-Keuls as a post-test, using the GraphPad Prism version 4.00 system (GraphPad Software, San Diego, CA, USA).

## Results

WKY and SHR diabetic or non-diabetic rats presented the general characteristics expected for these models, which are shown as supplementary material (Additional file 1: Table S1). Part of the general parameters of this study was presented previously [10]. SHR were hypertensive, and diabetes did not change this feature.

Salivary secretion (Table 1) was reduced in SHR, and diabetes induced an additional reduction. Salivary secretion was also reduced by diabetes in WKY rats. The salivary glucose concentration (Table 1) was lower in SHR than in WKY, an aspect never described before, and, as expected, diabetes increased it in both SHR-D and WKY-D.

**Table 1:** Morphological and functional characteristics of salivary glands of Wistar Kyoto (WKY) rats, diabetic WKY (WKY-D), spontaneously hypertensive rats (SHR) and diabetic SHR (SHR-D)

	**WKY**	**WKY-D**	**SHR**	**SHR-D**
Parotid weight (mg)	156 ± 5.6	101 ± 6.1***	138 ± 4.6#	124 ± 4.2##
Submandibular weight (mg)	288 ± 7.8	245 ± 13.8*	286 ± 7.2	234 ± 8.4**
Absolute salivary secretion (μL/7 min)	14.4 ± 0.6	6.7 ± 0.40***	8.0 ± 0.81###	3.5 ± 0.18***###
Relative salivary secretion (μl/g tissue/min)	4.6 ± 0.05	2.7 ± 0.06***	2.7 ± 0.07###	1.4 ± 0.02***###
Basal salivary glucose (mg/dL)	6.5 ± 0.32	23.4 ± 1.7***	3.2 ± 0.30#	16.4 ± 1.3***###

Relative salivary secretion was calculated taking into account the sum of parotid and submandibular weights. Data are means ± S.E.M of 5 animals. *P < 0.05, **P < 0.01 and ***P < 0.001 *vs* respective non-diabetic group; #P < 0.05, ##P < 0.01, ###P < 0.001 vs respective normotensive (WKY) group; One-Way ANOVA, Student-Newman-Keuls post-test.

**Figure 1:**
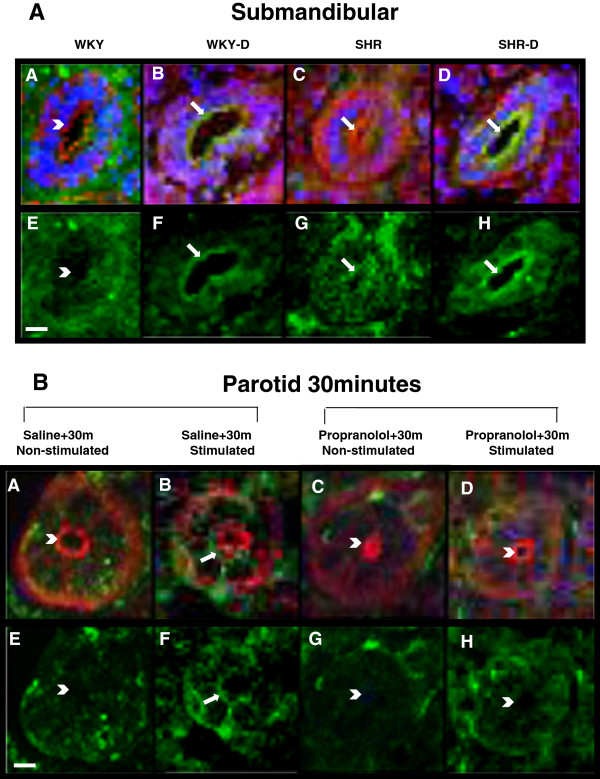
**Immunolocalization of the SGLT1 protein in ductal cells of salivary glands. A**. SGLT1 protein in ductal cells of submandibular glands of Wistar Kyoto rats (WKY), diabetic WKY (WKY-D), spontaneously hypertensive rats (SHR) and diabetic SHR (SHR-D). *A* to *D*: SGLT1 (green), F-actin (red) and nuclear marker (blue). *E* to *H*: only SGLT1 in green color. The SGLT1 protein in ductal cells of WKY can be seen in a very low intensity (*A* and *E*), whereas the SGLT1 immunoreactivity is clearly observed in WKY-D (*B* and *F*) and SHR (*C* and *G*); a further increase in SGLT1 can be observed in SHR-D (*D* and *H*). Arrowheads and arrows indicate the absence or presence of the SGLT1 protein in the luminal membrane of ductal cells; respectively. Scale bar, 20 μm. Images are representative of 4 animals in each group. **B**. SGLT1 protein in ductal cells of the parotid glands from Wistar-Kyoto rats that received physiological saline (*A*, *B*, *E* and *F*) or propranolol (*C*, *D*, *G* and *H*), and were subjected to 30-min sympathetic stimulation (*B*, *F*, *D* and *H*) or not (*A*, *E*, *C* and *G*). *A* to *D*: SGLT1 (green), F-actin (red) and nuclear marker (blue); *E* to *H*: only SGLT1 in green color. Scale bar, 20 μm. Arrowheads and arrows indicate the absence or presence of the SGLT1 protein in the luminal membrane of ductal cells; respectively. Images are representative of 4 animals in each group.

Figure 1A shows the presence of the SGLT1 protein in ductal cells of the submandibular gland, as well as its subcellular localization. In glands from WKY rat, immunofluorescence does not show important staining in the luminal membrane of ductal cells (*A* and *E*). However, in glands from diabetic (*B* and *F*) and hypertensive (*C* and *G*) rats, the SGLT1 protein is clearly observed in the luminal membrane of ductal cells. The association of diabetes and hypertension induced an additional expression of the SGLT1 protein in the luminal membrane, as well as a diffuse intracellular expression (*E*-*H*). Similar result was observed in the parotid gland (Additional file 2: Figure S1).

To verify the sympathetic activity role upon the SGLT1 protein translocation from the intracellular to the luminal membrane of ductal cells, we performed immunohistochemical analysis of non-stimulated and sympathetically stimulated parotid glands of non-diabetic WKY rats, subjected or not to previous β-blockade with propranolol (Figure 1B). In non-stimulated parotid glands, immunofluorescence does not reveal the SGLT1 protein in the luminal membrane of ductal cells (*A* and *E*), as described above. After 30 minutes of sympathetic stimulus, the SGLT1 protein content increased in the luminal membrane, demonstrating that the sympathetic activity induced the SGLT1 translocation (*B* and *F*). In basal (non-stimulated) parotid glands of propranolol-treated rats (*C* and *G*), only a low intracellular spread staining is observed, indicating that the β-blockade reduced the basal expression of the SGLT1 protein. Additionally, in the parotid glands from propranolol-treated rats subjected to sympathetic stimulus, the SGLT1 staining remained diffuse intracellularly, and did not appear near the luminal membrane of ductal cells (*D* and *H*).

## Discussion

In the present study, we demonstrate changes in the SGLT1 protein expression in salivary glands which can explain alterations in the salivary secretion observed in diabetic and hypertensive subjects. Considering the high capacity of SGLT1 to transport water [8], the increase in this protein at the luminal membrane of ductal cells observed in diabetic and hypertensive rats is in accordance with the decreased salivary flow rate detected in these animals. Thus, the inversely proportional regulation of the luminal SGLT1 content and of the salivary flow points out that this transporter is involved in the pathophysiology of diabetic and hypertensive xerostomia. In the acinar cells of salivary glands, where the SGLT1 protein plays a role in the cellular glucose disposal, similar regulations have been described in diabetic and hypertensive rats [10]. However, changes in the acinar SGLT1 expression do not contribute to the final salivary glucose concentration and salivary flow, as much as the changes in the luminal content of ductal cell shown here do.

Participation of the β-adrenergic activity in the SGLT1 expression and localization has already been described in acinar cells [10], and now, for the first time, we demonstrate the β-adrenergic role in the SGLT1 expression and translocation to the luminal membrane of ductal cells, where it must play a fundamental role in water reabsorption and; consequently; in the salivary flow.

It is important to point out that hypertensive rats (SHR) have a high sympathetic drive to the salivary glands [10], and this can explain the increased luminal content and cellular expression of SGLT1 observed in these animals. However, diabetes decreases the sympathetic activity in the salivary glands [10], but the luminal content of SGLT1 is increased in glands from both WKY and SHR diabetic rats, which implies an additional mechanism of regulation of the SGLT1 translocation. In this respect, the expression of a sugar sensor, which, via a G-protein-coupled receptor (GPCR), enhances SGLT1 translocation into the luminal membrane, was reported in the epithelial cells from the proximal intestine [13,14] and pancreatic ducts [15]. Considering that this glucose–sensing GPCR system might be expressed in the luminal membrane of ductal cells of salivary glands, high glucose concentration in primary saliva of diabetic rats could, therefore, explain the increased SGLT1 protein translocation, in spite of the decreased sympathetic activity. However, it is important to point that, although diabetes decreases the salivary gland sympathetic activity, in hypertensive rats it is still elevated as compared with normal rats [10]. Thus, the additional SGLT1 staining in the luminal membrane observed in ductal cells of diabetic SHR could be a consequence of a dual activation of PKA: by the high sympathetic activity and by the high luminal glucose concentration.

Glucose concentration in the primary saliva is closely related to plasma glucose concentration; nevertheless, glucose concentration in the final saliva of diabetic subjects has not been significantly correlated to their hyperglycemia [16,17]. In the present study, data from hypertensive rats point out the functional role of SGLT1 in the luminal membrane of ductal cells as a modulator of salivary glucose xconcentration. In SHR, the luminal SGLT1 increased in ductal cells, and the salivary glucose concentration decreased, as compared to WKY, in spite of the similar plasma glucose concentration, and the reduced salivary volume. On the other hand, in diabetic WKY and SHR, the glucose concentration was high in the final saliva, despite the expected increase in glucose reabsorption due to increased SGLT1 in the luminal membrane. This is a reasonably expected result, and can be compared to that observed in renal tubular glucose handling of diabetic subjects, in which urinary glucose increases, despite the increased glucose reabsorption by increased SGLT2 expression [18]. That occurs because glucose concentration in the glomerular filtrate is very high. At a glance, we are proposing that in diabetic rats, when plasma glucose concentration increases, the glucose concentration in the primary saliva also increases, and despite an increased ductal reabsorption, the glucose concentration in the final saliva will remain high.

In summary, we are reporting that the SGLT1 protein in the luminal membrane of ductal cells increases in salivary glands from WKY-D, SHR and SHR-D rats, inversely proportional to the non-stimulated salivary flow, demonstrating the important water reabsorption role of SGLT1 under these conditions. Furthermore, the luminal membrane expression of SGLT1 in ductal cells increases after sympathetic stimulus, which does not occur with previous β-adrenergic blockade, evincing the sympathetic-mediated regulation of SGLT1 translocation into the luminal membrane. This diabetes- and/or hypertension-induced increase in the ductal luminal SGLT1 protein, by increasing salivary water reabsorption, may explain the xerostomia reported by diabetic and hypertensive patients. The present study, by pointing out the role of SGLT1 in the salivary water reabsorption, paves the way for the future use of SGLT1 inhibitors in the treatment of diabetic-and hypertensive-induced xerostomia.

## Competing interests

All authors declare that they have no competing interests.

## Authors’ contributions

RSS proposed the research hypothesis, collected the data, and wrote the manuscript. MMO, ADS, RCM and HSF assisted in proposing the research hypothesis, collecting the data and writing the manuscript. UFM reviewed and edited the manuscript. All authors read and approved the final manuscript.

## Supplementary Material

Additional file 1: Table S1 Body weight, 24-hour urinary glucose excretion, plasma glucose, mean arterial pressure (MAP) and heart rate (HR) of Wistar Kyoto rats (WKY), diabetic WKY (WKY-D), spontaneously hypertensive rats (SHR) and diabetic SHR (SHR-D). Click here for file

Additional file 2: Figure S1 Immunolocalization of SGLT1 protein in ductal cells of parotid glands of Wistar Kyoto rats (WKY), diabetic WKY (WKY-D), spontaneously hypertensive rats (SHR) and diabetic SHR (SHR-D). *A* to *D*: SGLT1 (green), F-actin (red) and nuclear marker (blue). *E* to *H*: only SGLT1 in green color. SGLT1 protein in ductal cells of WKY can be seen in a very low intensity (*A* and *E*), whereas the SGLT1 immunoreactivity is clearly observed in WKY-D (*B* and *F*) and SHR (*C* and *G*); a further increase in SGLT1 can be observed in SHR-D (*D* and *H*). Arrowheads and arrows indicate absence or presence of SGLT1 protein in luminal membrane of ductal cells; respectively. Scale bar, 20 μm. Images are representative of 4 animals in each group. Click here for file
