# Supplementary material for: Increased SGLT1 expression in salivary gland ductal cells correlates with hyposalivation in diabetic and hypertensive rats
Source: Diabetol Metab Syndr. 2013 Oct 24;5:64. doi: 10.1186/1758-5996-5-64 (PMC4029169; doi:10.1186/1758-5996-5-64)
Supplement: Additional file 1: Table S1 — Body weight, 24-hour urinary glucose excretion, plasma glucose, mean arterial pressure (MAP) and heart rate (HR) of Wistar Kyoto rats (WKY), diabetic WKY (WKY-D), spontaneously hypertensive rats (SHR) and diabetic SHR (SHR-D). [file 1758-5996-5-64-S1.doc]

Table S1: Body weight, 24-hour urinary glucose excretion, plasma glucose, mean arterial pressure (MAP) and heart rate (HR) of Wistar Kyoto rats (WKY), diabetic WKY (WKY-D), spontaneously hypertensive rats (SHR) and diabetic SHR (SHR-D).

|  | WKY | WKY-D | SHR | SHR-D |
| --- | --- | --- | --- | --- |
| Body weight  (g) | 302 ± 6.1 | 206.9 ± 8.3*** | 311 ± 9.1 | 232 ± 8*** |
| Urinary glucose  (mg/24h) | 0.19 ± 0.07 | 206 ± 26*** | 1.9 ± 0.7 | 192 ± 22*** |
| Plasma glucose  (mg/dL) | 126 ± 13 | 304 ± 17*** | 134 ± 7.0 | 289 ± 9.5*** |
| MAP  (mmHg)  HR  (beats/min) | 120.5 ± 1.5  356 ± 6.0 | 118 ± 1.7    320 ± 6.5* | 172 ± 4.1###    439 ± 4.8### | 165 ± 6.8###    425 ± 17.9### |

Data are means ± S.E,M, of 5 animals. *P<0.05 and ***P<0.001 *vs* respective non-diabetic group; ###P<0.001 vs respective normotensive (WKY) group; One-Way ANOVA, Student-Newman-Keuls post-test.
