# Supplementary material for: Increased SGLT1 expression in salivary gland ductal cells correlates with hyposalivation in diabetic and hypertensive rats
Source: Diabetol Metab Syndr. 2013 Oct 24;5:64. doi: 10.1186/1758-5996-5-64 (PMC4029169; doi:10.1186/1758-5996-5-64)
Supplement: Additional file 2: Figure S1 — Immunolocalization of SGLT1 protein in ductal cells of parotid glands of Wistar Kyoto rats (WKY), diabetic WKY (WKY-D), spontaneously hypertensive rats (SHR) and diabetic SHR (SHR-D). A to D: SGLT1 (green), F-actin (red) and nuclear marker (blue). E to H: only SGLT1 in green color. SGLT1 protein in ductal cells of WKY can be seen in a very low intensity (A and E), whereas the SGLT1 immunoreactivity is clearly observed in WKY-D (B and F) and SHR (C and G); a further increase in SGLT1 can be observed in SHR-D (D and H). Arrowheads and arrows indicate absence or presence of SGLT1 protein in luminal membrane of ductal cells; respectively. Scale bar, 20 μm. Images are representative of 4 animals in each group. [file 1758-5996-5-64-S2.ppt]

## Slide 1
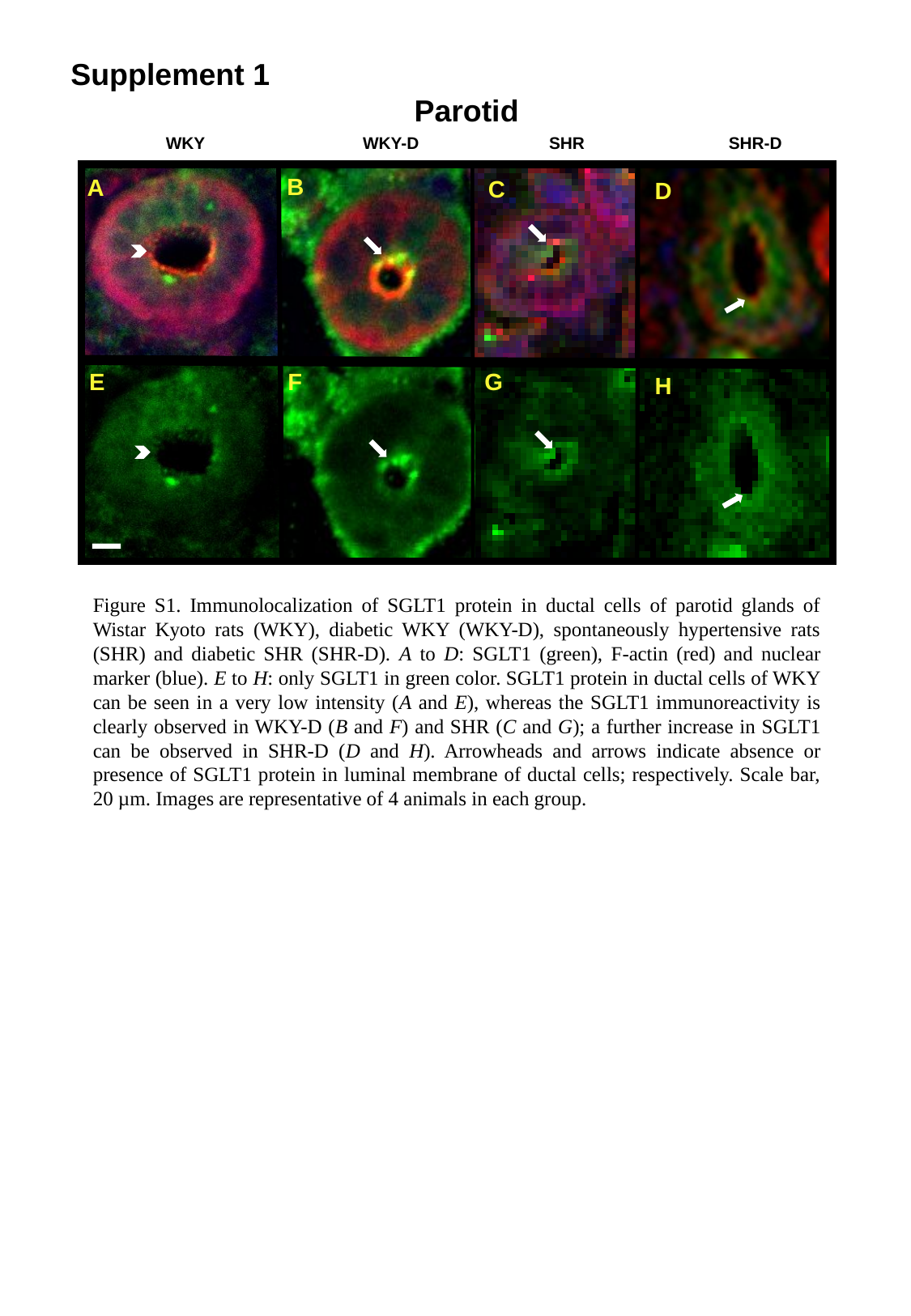

Supplement 1
Parotid
 WKY WKY-D SHR SHR-D
B
A
C
D
E
F
G
H
Figure S1. Immunolocalization of SGLT1 protein in ductal cells of parotid glands of Wistar Kyoto rats (WKY), diabetic WKY (WKY-D), spontaneously hypertensive rats (SHR) and diabetic SHR (SHR-D). A to D: SGLT1 (green), F-actin (red) and nuclear marker (blue). E to H: only SGLT1 in green color. SGLT1 protein in ductal cells of WKY can be seen in a very low intensity (A and E), whereas the SGLT1 immunoreactivity is clearly observed in WKY-D (B and F) and SHR (C and G); a further increase in SGLT1 can be observed in SHR-D (D and H). Arrowheads and arrows indicate absence or presence of SGLT1 protein in luminal membrane of ductal cells; respectively. Scale bar, 20 µm. Images are representative of 4 animals in each group.
